# Effectiveness of nursing intervention for increasing hope in patients with cancer: a meta-analysis^1^

**DOI:** 10.1590/1518-8345.1920.2937

**Published:** 2018-08-09

**Authors:** Ping Li, Yu-Jie Guo, Qing Tang, Lei Yang

**Affiliations:** 2 MSc, Researcher, School of Nursing, Nantong University, Nantong, Jiangsu, China.; 3 PhD, Assistant Professor, School of Nursing, Nantong University, Nantong, Jiangsu, China.

**Keywords:** Neoplasm, Hope, Meta-Analysis

## Abstract

**Objective:**

to evaluate the efficacy of nursing interventions to increase the level of hope in cancer patients, in a meta-analysis.

**Methods:**

electronic databases were searched. Two of the authors independently extracted data from the eligible studies, and Stata 13.0 software was used to pool the data.

**Results:**

nine randomized controlled trials were included, and methodological quality of each randomized controlled trial (RCT) was evaluated using Cochrane handbook recommendations. A random effects model was used to combine results from eligible studies. The pooled results using the fixed effects model showed that scores to first effects increase significantly after the use of nursing intervention between the groups. Heterogeneity was observed among the studies for posttest (df = 8, P = 0.000; I^2^ =76.1 %). The results indicated significant heterogeneity across the nine selected studies. The test for heterogeneity showed no homogeneity among studies for follow-up (df = 8, P = 0.328; I^2^ = 12.9 %), and there was no statistical significance.

**Conclusion:**

the current evidence suggests that nursing intervention has a positive effect on hope in cancer patients. However, more large-scale and high-quality randomized controlled trials are needed to confirm these results.

## Introduction

Hope has been defined as the possibility of a better future in the context of uncertainty^1^, which significantly increases a patient’s quality of life^2^. It has been identified as a valuable psychological resource that enables the individual to take an interest in his/her life and future, and to find meaning in life^3^. The author^4^ ^)^ stated that the most important feature of hope is that it gives confidence to the individual to make life changes.

It is well known that the cancer diagnosis, its treatment, and the challenges of survivorship increase patients’ levels of psychological symptoms to a degree that might affect their adaptation to their disease^5^. Nursing intervention has been shown to improve hope through promoting greater psychological wellbeing and decreasing psychological problems, such as depression and anxiety^5^ ^-^ ^6^.

Cancer diagnosis and treatment can affect physical functioning, mental health, and quality of life of individuals with cancer^7^. A great deal of studies^8^ ^-^ ^9^ ^)^ have shown that the long-term and late effects following a cancer diagnosis have an impact on patients, including functional deficits, mood disturbances and heart failure in relation to chemotherapy toxicity. Many of these factors influence patients’ hope, which has been considered an important coping strategy among cancer patients. Many researchers^10^ ^-^ ^11^ ^)^ found that a high level of hope was associated with lower levels of anxiety and depression, higher social support, and better quality of life.

Several studies have shown that the influence of healthcare professionals has great potential to effect hope among cancer patients. One study^12^ ^)^ evaluated a psychologically supportive intervention, based on the theory called “Transforming hope”, in which patients were guided to view a film on hope and work on a hope activity. Higher hope and quality of life among cancer patients were found in patients after the intervention. Another study^13^ ^)^ found a novel treatment intervention combining three central attributes of mindfulness, hope therapy, and bio-behavioral components which were provided to women with cancer recurrence. That intervention increased hope and mindfulness two, four and seven months after the intervention. However, the effectiveness of nursing interventions for enhancing hope among cancer patients remains controversial. The author^14^ ^)^ found that exercise leads to a great improvement in strength among lung cancer patients, but not hope. One researcher^15^ ^)^ studied the effects of telephone intervention led by nurses, and found no clear difference in the level of hope among patients during chemotherapy.

From the nursing point of view, helping patients experiencing difficult situations to maintain hope is an essential goal in providing care to patients who are struggling with a diagnosis of cancer. In addition, previous studies have used various types of nursing intervention, which hinders the determination of whether nursing intervention foster hope in cancer patients.

Therefore, it is necessary to summarize the results from randomized clinical trials to assess the efficacy of nursing intervention to improve hope in cancer patents. To examine this hypothesis, we conducted the meta-analysis, and assumed that nursing intervention has a beneficial effect on hope in patients with cancer.

## Methods

The Preferred Reporting Items for Systematic Reviews and Meta-Analyses (PRISMA) guidelines, issued in 2009, was utilized to report this meta-analysis^16^. Relevant studies were identified through systematic searches of the electronic databases, from their inception until January of 2016.We searched the Cochrane Library databases, PubMed, Ovid, Web of Science, China National Knowledge Infrastructure (CNKI), and Wanfang Data for articles published. Any randomized controlled study that evaluated the association between nursing intervention and the level of hope in adult patients with cancer was eligible for inclusion in our study, and no restrictions were placed on language or publication status. Both Medical Subject Headings (MeSH) terms, and the keywords of “cancer OR neoplasm”, “hope”, “nurse-led OR nurse” AND “randomized controlled trial OR controlled clinical trial” were used as search terms. Additionally, we scanned the reference lists of retrieved papers for any additional relevant studies. We also contacted the corresponding author or first author to obtain information if publications were unclear or more information was needed.

Studies were eligible for inclusion in the present meta-analysis if they met the following criteria: (a) randomized control trial design; (b) included only adult cancer survivors (age >18); (c) compared nursing interventions with usual care; (d) authors reported effective hope scores and 95 % confidence intervals (CIs) on outcomes for comparisons.

Studies that assessed the hope outcome using validated scales (e.g., Herth Hope Index - HHI). The Herth Hope Index (HHI) contains 12 items that measure three dimensions of hope^17^. The HII delineated three factors of hope: a) temporality and future, b) positive readiness and expectancy, and c) interconnectedness^18^. Each item is rated on a 4-point Likert scale that ranges from “strongly disagree (1)” to “strongly agree (4)”. A total HHI score that can range from 12 to 48 is calculated, and higher scores indicate higher levels of hope. It has been used successfully in studies with persons with cancer and their family caregivers^19^. The Chinese version of HHI has demonstrated the test-retest reliability, internal consistency, content validity and construct validity in cancer patients^20^.

However, if the study provided no original data, or insufficient information on hope, it was excluded. Publications that were letters, comments, correspondence, editorials, reviews, or gray literature were not eligible. If the study involved caregivers of cancer patients, it was excluded. Two investigators independently screened the abstracts or full-text articles identified, using the search strategy previously described, to assess the eligibility of studies in a standardized manner.

Based on the detailed data of the included studies, two reviewers independently evaluated the quality of eligible trials using the assessment tool described in the Cochrane Handbook for Systematic Reviews of Interventions. Parameters of risk of bias were graded as high, low, or unclear. The following domains were assessed in relation to their risk of bias: random sequence generation; allocation concealment; blinding (performance bias, detection bias); incomplete outcome data (attrition bias); selective reporting (reporting bias); and other sources of bias^21^. Any discrepancy was resolved by consultation, or adjudicated by a third reviewer serving as the arbitrator.

Data from each study were independently extracted by the two investigators. Any disagreements were resolved by a third reviewer. Information abstracted from each study included the first author, year of publication, country, age at baseline, sample size, follow-up duration, characteristics of the intervention (e.g. type, frequency, length), primary outcomes measure. Discrepancies were rechecked by the corresponding author of the current article and consensus was achieved by discussion.

Continuous variables were analyzed using standardized mean difference (SMD) and expressed with 95% confidence intervals (CI); random effects methods were only reported when the heterogeneity among the combined study results was statistically significant, by evaluating the Cochran Q and the I^2^ statistic, with p < 0.05 indicating significant heterogeneity^22^. A p-value for Cochrane’s Q test at < 0.1 with an I^2^ value > 50% indicated no or slight heterogeneity across studies, and then a fixed-effect model was applied; otherwise, a random-effect model was adopted to pool the data^23^. If the results were presented as median and range values, the means and standard deviation were calculated using the formulas^24^. Subgroup analyses were conducted by dividing the studies into groups according to (a)sex, (b) type of cancer, (c) whether hope was the primary outcome, (d) quality of included study, (e) intervention format, and (f) intervention providers. Potential publication bias was evaluated using Begg^25^ funnel plots and Egger^26^ ^)^ tests. Two-tailed p-value < 0.05 was considered statistically significant. In view of the significant heterogeneity among the studies included in our meta-analysis, sensitivity analysis was performed by removing the individual study with the largest effect size to assess whether the results could have been affected markedly by a single study. The Stata 13.0 (StataCorp, College Station, TX) statistical software was applied to pool the results in this meta-analysis.

## Result

The literature search initially yielded 1119 relevant articles, after a comprehensive search. Citation search identified another 13 articles. Of the publications, 534 duplicate articles were excluded. After screening the title and abstract using the inclusion and exclusion criteria, 589 articles were removed. Ultimately, the remaining nine randomized clinical trials^2^ ^,^ ^27^ ^-^ ^34^ involving participants were included in the meta-analysis.

## Characteristics of Included Studies

**Figure 1:**
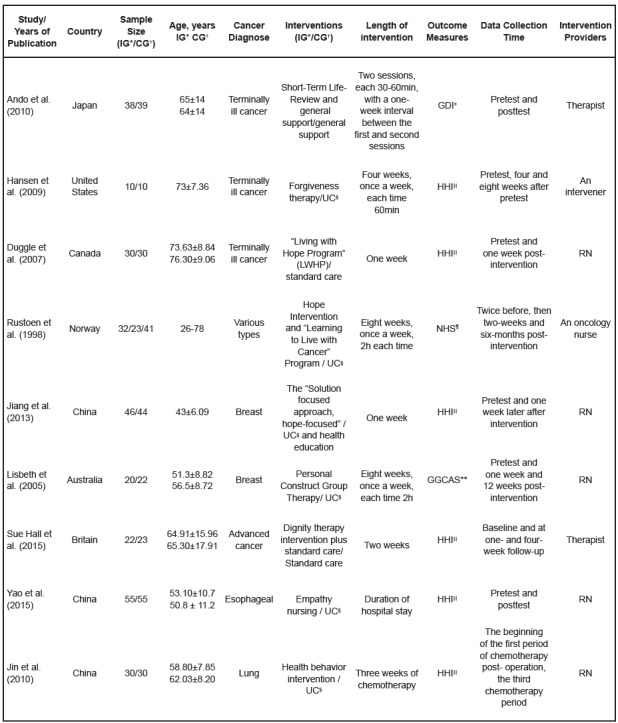
Characteristics of randomized controlled trials of participants and interventions. Nantong, Jiangsu province, China, 2016

**Figure 2:**
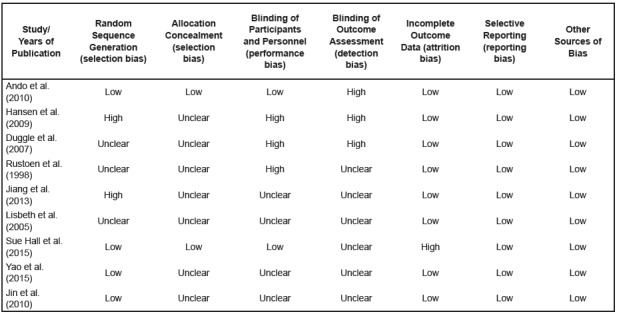
Summary of Cochrane’s Risk of Bias. Nantong, Jiangsu province, China, 2016

Some details of the included studies are presented in Figure 1. Study sample sizes ranged from 20 to 116. Of a total population of 600 randomized patients, 306 were in the intervention group, and 294 in the control group. The randomized controlled trails were published between 1998 and 2015. Of them, four studies were conducted in Asia (one in Japanese^27^ and three in China^30^ ^,^ ^33^ ^-^ ^34^ ), two in Europe^2^ ^,^ ^32^, one in the USA^28^, one in Canada^29^ and one in Australia^31^. All studies included one control group, and the control group was treated with usual care. However, there was an article that was divided into three groups, with the inclusion of an additional intervention named an attention control group. The most common treatment format was an individual approach (n=7), and only two studies applied a group approach. The most frequently used hope measurement was the HHI. In nine studies, there were various interventions considered. Most interventions were provided in hospitals or in patients´ homes. Among the nine studies, interventions were delivered by health personnel (e. g., a nurse) in six studies, and other professionals were the interventionists in three studies. The mean length of intervention was 3.2 weeks. The mean total intervention time was 86.5 minutes, with total intervention time in each study ranging from 30 to 120 minutes. The quality assessment of included studies, using the risk of bias tool, is shown in Figure 2. Overall, one randomized controlled trial had a score of 13^27^, one trial had a score of 11^32^, one trial had a score of 9^28^, three trials had a score of 8^29^ ^,^ ^33^ ^-^ ^34^, two trials had a score of 7^2^ ^,^ ^30^, and the remaining one trial had a score of 6^31^. The mean score was 8.5, suggesting a moderate quality of the reports included in this meta-analysis. Among all the selected studies, participants and personnel were mostly not double blinded. Outcome assessment was not blinded in any of the studies. Overall, all the included studies were considered to have a high risk of bias.

*Intervention Group, †Control Group, ‡The Good Death Inventory, §Usual Care, ||Herth Hope Index, ¶Nowotny Hope Scale, **Gottschalk-Gleser Content Analysis

## Nursing Intervention on Hope

**Figure 3:**
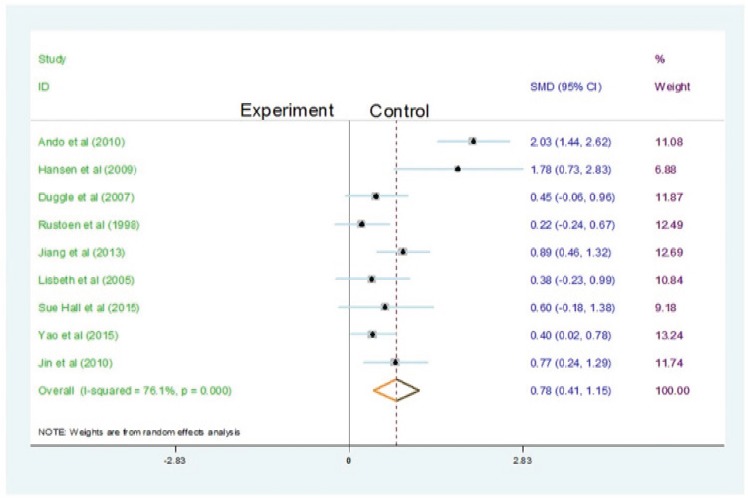
The efficacy of nursing intervention on hope from baseline to posttest

**Figure 4:**
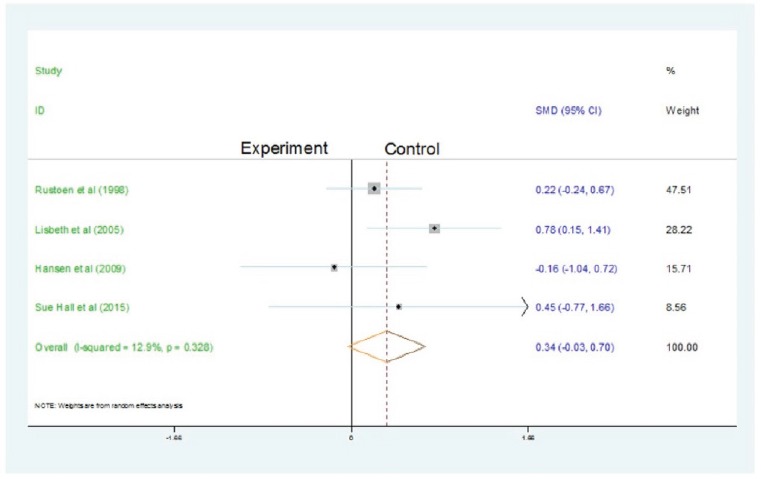
The results of nursing interventions on hope, from baseline to follow-up

Figure 3 presents the efficacy of nursing interventions on hope, from baseline to posttest, and the differences between intervention and control groups are estimated. The pooled results from the included studies indicated that nursing intervention contributed to a significant enhancement in hope, when compared with the control treatment. Figure 4 summarizes the results of nursing interventions on hope, from baseline to follow-up. The pooled results using the fixed effects model showed that scores to first effects increased significantly after the use of nursing intervention between the groups. Heterogeneity was observed among the studies for post-test (df = 8, p= 0.000; I^2^ =76.1 %). The results indicated significant heterogeneity across the nine selected studies. The test for heterogeneity showed no homogeneity among studies for follow-up (df = 8, p = 0.328; I^2^ = 12.9 %), and there was no statistical significance.

## Subgroup Analysis

Table 3 presents the results of subgroup analyses of sex, type of cancer, whether hope was the primary outcome, research quality, intervention format and intervention providers. In stratified analyses, differences between males and females were statistically significant (SMD= 0.83; 95%CI= 0.35-1.32). The effect sizes of studies in which hope was the secondary outcome (SMD= 1.18; 95%CI= 0.29-2.07) were statistically significant. Nursing intervention significantly improved hope in individuals with terminal cancer (SMD= 1.39; 95%CI= 0.25-2.53). In subgroup analyses by intervention format, an individual approach across seven studies showed significant effects on hope (I^2^=77.6%, 95%CI=0.49,1.38, *p*=0.000). Group therapy was evaluated in two trials, and showed no significant differences in hope (I^2^=0.0%, 95%CI=-0.09,0.64, *p*=0.670). In subgroup analyses performed by intervention providers, six studies provided by health personnel showed significant effects on hope (I^2^=17.5%, 95%CI=0.30,0.73, *p*=0.300). In contrast, three studies conducted by other professionals also indicated significant differences in hope (I^2^=76.5%, 95%CI=0.54,2.41, *p*=0.014).

**Table 3:** Overall Results and Subgroup Analyses of Nursing Intervention on Hope. Nantong, Jiangsu province, China, 2016

Subgroups	No. of studies	SMD*	95%CI^†^	I^2‡^ %	*p* Value
Overall	9	0.78	0.41-1.15	76.1	0.000
Sex					
Female	2	0.68	0.19-1.17	43.5	0.183
Male and female	7	0.83	0.35-1.32	81.0	0.000
Cancer type					
Breast cancer	2	0.68	0.19-1.17	43.5	0.183
Terminally ill cancer	3	1.39	0.25-2.53	88.3	0.000
Others	4	0.44	0.20-0.69	0.0	0.450
Hope as the primary outcome					
Yes	5	0.54	0.29-0.78	31.7	0.210
No	4	1.18	0.29-2.07	83.3	0.000
Quality of study					
Score > 8	3	1.48	0.54-2.41	76.5	0.014
Score < 8	6	0.52	0.30-0.73	17.5	0.300
Intervention format					
Individual approach	7	0.93	0.49-1.38	77.6	0.000
Group therapy	2	0.28	-0.09-0.64	0.0	0.670
Intervention providers					
Health personnel	6	0.52	0.30-0.73	17.5	0.300
other professionals	3	1.48	0.54-2.41	76.5	0.014

*Standardized Mean Difference, †Confidence Interval, ‡Inconsistency

## Sensitivity Analysis

Given the heterogeneity among the studies for our finding, sensitivity analysis was performed by excluding an individual study, and the data of the remaining studies were chosen and pooled. After excluding the lowest study score^31^, the result did not change significantly (SMD= 0.83; 95%CI= 0.42-1.24).

## Discussion

With increasing pressure on emotional changes, and the need to improve care worldwide, nursing interventions to increase levels of hope are of significant importance. Hope is the most common psychological factor after diagnosis, and is a major contributing factor to quality of life. However, evidence of the benefits of nursing interventions on hope in cancer patients is rarely presented. We conducted this meta-analysis, including nine randomized controlled trials, to evaluate the effect of nursing intervention on hope in cancer patients.

Overall, the findings from our study indicated that nursing interventions can significantly improve the level of hope among cancer patients. Caring behaviors by nurses have been suggested to maintain and foster hope in patients with cancer. Furthermore, the mechanism by which nursing intervention could influence the level of hope in cancer patients is that nurses encourage patients with cancer to construct and rebuild appropriate strategies to enhance hope. Additionally, nursing interventions may help patients find meaning and purpose within a life-threating illness, dictate their ability to cope with the disease in a meaningful way, and provide for the needs of cancer patients^35^.

## According to clinical characteristics

According to the result of subgroup analyses by sex, males and females showed a significant effect on hope. Similar to one study, the author did a comparison to explore the relationship between urban or rural background and health attitudes of newly diagnosed oncology patients, which demonstrated that males scored significantly higher for belief^36^. There is a need to carry out more well-designed studies to verify our conclusion.

In subgroup analyses by type of cancer, a significantly higher level of hope was noted in individuals with terminal cancer than in other cancers, when using nursing interventions. This effect was not found for two trials with breast cancer patients and four trials with other cancers. The result is consistent with another study in this field^37^. However, more RCTs on various types of cancer will be needed to confirm our conclusion.

## According to intervention characteristics

The finding from this meta-analysis based on 600 study participants indicated that nursing interventions have a positive influence on hope, and the positive effects were consistent either posttest or through follow-up, or both. The lengths of interventions for most studies included in this meta-analysis were less than eight weeks. This result is meaningful, and it is in accordance with that of previous meta-analysis studies. The researchers^38^ aimed to identify whether interventions can reduce emotional distress in patients and their caregivers. Based on 29 randomized clinical trials, the author concluded that the average dose of the interventions was 6.7 sessions. The findings from our study support the hypothesis that nursing intervention can significantly increase hope in cancer patients. Participants who were exposed to intervention designed to increase the feeling of hope had higher hope scores than those who were not exposed to intervention apart from regular care and hospital follow-up.

In subgroup analyses, according to intervention format, the results show that individual therapy is better than group therapy in cancer patients. Even if group approach interventions were effective in some aspects, the current results are in accordance with those of previous meta-analysis in concluding that psychosocial interventions using individual treatments (n=4) were more effective in increasing survival time than group intervention (n=11)^39^. There are only two articles using group therapy, which are too few. Therefore, further study for intervention format will be essential in the future.

## Implication for research

Some of the evidence on the effectiveness of nursing intervention on hope domains reported in this article find support in the literature^40^. However, some differences exist when comparing findings with other reviews, because other reviews included healthy or unhealthy people. Similar to other reviews, the authors documented positive effects of nursing interventions on hope. Variations in findings reported by the reviews could be explained by differences in inclusion criteria, treatment status, duration of the nursing intervention, and measures used to assess hope. Several areas for future research can improve understanding of the effects of nursing interventions on hope, and there also is a need to understand the necessary frequency, duration and format of nursing interventions for optimal and sustainable effect.

Because of the character of hope, a dynamic yet multidimensional psychological resource, most scholars tend to do qualitative research. The authors^41^ provided a meta-synthesis of qualitative research on the hope experience of older persons with chronic illness; twenty relevant published articles were included. Findings indicated that the concept of hope differs for older and younger adults experiencing suffering. In addition, resources for hope are both internal and external. Another systematic review was conducted on positive psychology interventions in breast cancer^42^. Based on 16 studies, which synthesized the evidence about the positive psychology interventions, the result showed that hope was one of the five groups of therapies in structuring positive psychology. Family caregivers (FCs) are involved in all aspects of patient care. To explore the information about FCs' levels of hope, a recent cross - sectional study found that family caregivers of persons with advanced cancer have a lower level of hope, associated with a higher level of caregiver role strain^43^. These findings suggest that some populations could be prioritized in public mental health interventions to prevent the occurrence of hopelessness, and interventions need to be provided to enhance hope.

This review identified several beneficial effects of nursing interventions on hope. In addition, as evidence accumulates, research will become increasingly precise in identifying what kinds of nursing interventions benefit which cancer survivors. In the meantime, the current evidence supports the translation of the accumulated knowledge base to practice. The evidence reported in this article should help inform healthcare professionals, cancer survivors, and educators that nursing interventions have a beneficial effect on hope.

## Limitations

Most of the studies included in this meta-analysis involved individuals with breast and terminal cancers; additional RCTs that investigate the beneficial effects of nursing intervention on hope are warranted in individuals with different types of cancer . In addition, only one article in this meta-analysis revealed that nursing intervention significantly improved level of hope among individuals with cancer before, during, and after cancer treatment. It is known that cancer is a complex and heterogeneous disease, which is noted for marked global variations in etiology, incidence, and management^44^. Consequently, there might be a certain amount of clinical heterogeneity, even though we detected no statistical heterogeneity through our study. Meta-analysis is considered hypothesis-generating, and is not conducted to test a hypothesis or establish a standard of care^45^. Additionally, meta-analysis is a secondary study that is based on primary studies, and some bias is inevitable^46^. Fourth, the quality of meta-analysis is dependent on the quality and comparability of information from the primary studies. If individual information were available, a more precise analysis, such as individual patient data meta-analysis, should be conducted rather than conventional meta-analysis. This is a big project, and it needs authors of all published papers to share their data. Fifth, given that hopelessness is highly prevalent among cancer patients, greater emphasis should be placed on establishing nursing programs that increase access to mental health care, as well as for patients at different stages of their disease and treatment trajectory.

## Conclusions

Evidence from this study indicates that nursing interventions are certainly useful strategies in increasing hope with cancer. Health care providers must convey the effectiveness of nursing interventions to individuals with cancer who are facing problems with hope. Furthermore, stratified analyses suggested that patients with terminal cancers had a significantly increased CI of total hope level than any other cancer. Future studies should focus on specific populations. However, it is noted that more high-quality RCTs are needed to further confirm these findings.
